# Insulin-like growth factor binding protein-6 modulates proliferative antagonism in response to progesterone in breast cancer

**DOI:** 10.3389/fendo.2024.1450648

**Published:** 2024-12-04

**Authors:** Francisco J. Lariz, Pacha B. Botero, Isabella Shoffstall, Kevin D. Houston

**Affiliations:** New Mexico State University, Department of Chemistry and Biochemistry, Las Cruces, NM, United States

**Keywords:** breast cancer, progesterone, progesterone receptor, insulin- like growth factor binding protein, steroid hormones

## Abstract

Breast cancer is one of the most diagnosed cancers worldwide. The insulin-like growth factor (IGF) system promotes proliferation and survival in breast cancer cells and is regulated by 6 insulin-like growth factor binding proteins (IGFBPs). The IGFBPs sequester IGFs to prolong their half-life and attenuate binding to insulin-like growth factor 1 receptor (IGF1R). While IGFBP-6 has been studied in some cancers it has not been studied extensively in hormone receptor positive breast cancer. Survival analysis using available databases indicated that high IGFBP-6 levels improve overall survival in progesterone receptor positive breast cancers. IGFBP-6 is transcriptionally induced by progesterone in T47D breast cancer cells resulting in increased intracellular and extracellular IGFBP-6 protein. Knockdown of IGFBP-6 resulted in reduced proliferative antagonism when estradiol stimulated T47D cells were cotreated with progesterone and protein levels of both progesterone receptor isoforms (PR-A and PR-B) were decreased following knockdown of IGFBP-6. P21(Cip1/Waf1), which is progesterone responsive, was not induced in response to progesterone following knockdown of IGFBP-6. Cyclin E2, a cell cycle regulator, is induced by progesterone only when IGFBP-6 is knocked down. Stable overexpression of IGFBP-6 in MCF-7 cells resulted in an increase in Epidermal Growth Factor Receptor (EGFR) and this expression was further enhanced when cells were cotreated with progesterone and estradiol. These results indicate that IGFBP-6 is a regulator of progesterone action, and that PR is required for the observed protective effects of IGFBP-6 in breast cancer.

## Introduction

Insulin-like growth factor binding protein-6 (IGFBP-6) is one of a family of six insulin-like growth factor binding proteins (IGFBPs) which regulate the activity of the insulin-like growth factor 1 and 2 (IGF1 and 2) (1–3). IGFBPs sequester IGFs to attenuate binding to Insulin-like growth factor 1 receptor (IGF1R) which promotes proliferation and survival in breast cancer cells (4). IGFBPs have their own affinities for the IGFs, however IGFBP-6 has a 20-50-fold binding affinity for IGF-2 over IGF-1 (5). IGFBP-6 inhibits proliferation in cancer cells which are IGF-2 dependent and can function independently of IGF-2 to inhibit angiogenesis, senescence, and cell migration (6). However, the role of IGFBP-6 in breast cancer is not well defined.

Breast cancer is one of the most common cancers world-wide with over 2,000,000 new cases and over 626,000 deaths in 2018 (7, 8). Over 70% of breast cancers are hormone receptor positive meaning they express high levels of Estrogen Receptor Alpha (ERα) and Progesterone Receptor (PR) (9). PR is a transcriptional target and a biomarker of functional ERα (10). ERα and PR bind the ovarian steroid hormones Estradiol (E2) and Progesterone (P4), respectively (11–13). In ERα-positive breast cancer, E2 promotes proliferation and survival. (11, 14) while activated PR antagonizes the proliferative effects of estrogen receptor *in vitro* (13, 15–17). However, the role of progestogens and PR are not fully understood in breast cancer (18, 19). Despite the increase in breast cancer risk from synthetic progestins, progesterone, the endogenous progestogen, does not increase breast cancer risk (18, 20–22).

Progesterone receptors (PR) consist of multiple isoforms of which the most studied are PR-A and PR-B which are transcribed from the same gene (PGR) from alternate start codons (19, 23, 24). PR-B is a 110 kDa protein and PR-A a 90 kDa protein which lacks the first 164 N-terminal residues found in PR-B. (19, 23–25). PR-B is responsible for most of the transcriptional activity when compared to PR-A (23). PR-B can dimerize with another PR-B or bind with other nuclear receptors such as ERα to promote transcription of target genes (12). PR-B also binds with the SP-1 transcriptional complex to indirectly promote transcription of epidermal growth factor receptor (EGFR) and p21 (Cip1/Waf1) (26). EGFR is a receptor associated with proliferation, survival, and chemoresistance in breast cancer (27). P21 is a cell cycle inhibitor which binds cyclin dependent kinase 2 and cyclin dependent kinase 4/6 (28). PR-A has low transcriptional activity and is responsible for trans-repression of other nuclear receptors including ERα and PR-B (29–31). In a study which characterized transcriptomic changes in response to progestins, it was shown that treatment with P4 and E2 resembles treatment with P4 alone (32, 33). The presence of PRs in ER+ breast cancer is associated with improved survival compared to ER+, PR- breast cancer (33, 34).

In this study, survival analysis demonstrated that high IGFBP-6 improved overall survival in PR positive breast cancer patients. In T47D breast cancer cells, P4 induced IGFBP-6 transcriptionally and was inhibited by treatment with mifepristone (RU 486). Cell proliferation was measured following treatment of cells with P4, E2, or both. Cell proliferation increased with E2 but not P4 alone. Cotreatment with both E2 and P4 produced no change in proliferation relative to the control. SiRNA knockdown of IGFBP-6 had no significant effect on proliferation with P4 or E2 treatment alone but increased proliferation after cotreatment with both P4 and E2. This loss of proliferative antagonism in IGFBP-6 knockdowns was accompanied by decreases in both PR-B, PR-A, and p21 (Cip1/Waf1) following cotreatment of P4 and E2. Cyclin E2 which was not induced by P4 in the controls, was induced after IGFBP-6 knockdown. These results suggest that IGFBP6 is a modulator of progesterone’s antagonistic effects on breast cancer cell proliferation.

## Materials and methods

### Survival analysis

Kaplan-Meier survival plots were produced from data in the KM Plotter database (35). This database contains RNA-seq expression and Genechip microarray data from 55 datasets obtained from the European Genome-Phenome Archive (EGA) and the Gene Expression Omnibus (GEO). Overall survival was analyzed using RNA-Seq data and Genechip microarray data from EGA and GEO. Recurrence-free survival was analyzed using microarray data only. Analysis was conducted by selecting IGFBP-6 expression in PR positive patients.

### Cell culture

T47D breast cancer cells were acquired from ATCC (Manassas, VA). Cells were maintained in DMEM supplemented with 10% fetal bovine serum, 1mM sodium pyruvate, and 2mM L-glutamine (Life Technologies, Carlsbad, CA). Cells below passage 30 were used and all nucleotide and protein purifications were performed on cells at similar confluency.

### Steroid and antiprogestin treatments

Cells were treated with 10 nM estradiol (E2) (Millipore Sigma, St. Louis MO), 50 nM progesterone (P4) (Millipore Sigma, St. Louis MO), or 10nM E2 plus 50nM P4 in ethanol. Cells were also treated with 25 nM Mifepristone (RU 486) (Millipore Sigma, St. Louis MO) either alone or in the presence of 50nM P4. Cells were plated and given 24 hours to adhere to the plate, washed with 1X PBS and then treated in DMEM supplemented with 10% Charcoal-stripped FBS, 1mM sodium pyruvate, and 2mM L-glutamine (Life Technologies, Carlsbad, CA) plus the corresponding steroid or antiprogestin treatment.

### Total RNA extraction and quantitative real-time PCR analysis

Total RNA was extracted using the PureLink RNA Mini Kit (Life Technologies, Carlsbad, CA). Synthesis of cDNA was done with 1 μg of extracted RNA using the High-Capacity RNA-to-cDNA Kit (Life Technologies, Carlsbad, CA) and used as a template for quantitative real-time PCR (RT-qPCR) reactions. RT-qPCR was performed with SYBR Green Master Mix (Life Technologies, Carlsbad, CA) and the 7300 Real-Time PCR system (Bio-Rad, Hercules, CA). Human RPL30 was used as an internal control to normalize for mRNA in RT-qPCR reactions. The following primer pairs were used: IGFBP-6 (Forward 5’-CGAGGGGCTCAAACACTCTA-3’, Reverse 5’-CATCCGATCCACACACCAG-3’), and RPL30 (Forward 5’- ACAGCATGCGGAAAATACTAC-3’, Reverse 5’- AAAGGAAAATTTTGCAGGTTT-3’).

### Immunoblot analysis

This method has been described previously (36) Cells were lysed with RIPA buffer containing protease and phosphatase inhibitor cocktails (Life Technologies, Carlsbad, CA). After lysis cells were centrifuged at 9,000 x g for 20min at 4°C and the supernatant was collected. Protein concentrations were determined by BCA assay (Thermo Scientific, Rockford, IL). 10-30 μg of protein was resolved using Bolt 4-12% Bis-Tris Plus gels and dry transferred to a PVDF membrane with the iBlot2 system (Life Technologies, Carlsbad, CA). Membranes were blocked in 1X Tris-buffered saline 0.1% Tween 20 (TBST) and either 10% Bovine Serum Albumin (BSA) (Thermo Scientific, Rockford, IL) for phospho-proteins or 5% fat-free milk for all other protein targets. Membranes were then washed in 1X TBST three times, primary antibody was added and allowed to incubate overnight at 4°C. Proteins were incubated at a ratio of 1:1000 in 5% BSA in TBST for phosphoproteins or in 5% milk in TBST for other proteins. Antibodies used are as follows: IGFBP-6 (#ab219560, Abcam, Waltham, MA), Phospho-PR (#13736, Cell Signaling Technology, Danvers, MA), PR (#8757, Cell Signaling Technology, Danvers, MA), p21 (#2947, Cell Signaling Technology, Danvers, MA), EGFR (#4267, Cell Signaling Technology, Danvers, MA), Cyclin E1 (#4129, Cell Signaling Technology, Danvers, MA), Cyclin E2 (#4132, Cell Signaling Technology, Danvers, MA) and Beta Actin (#sc 47778, Santa Cruz Biotechnology, Dallas, TX).

After incubation with primary antibodies, membranes were washed three times with 1X TBST then incubated with either anti-rabbit IgG conjugated to horseradish peroxidase (#7074, Cell Signaling Technology, Danvers, MA) or anti-mouse IgG conjugated to horseradish peroxidase (#7076, Cell Signaling Technology, Danvers, MA) for 1hr at room temperature with a dilution ratio of 1:5000. Membranes were then washed three times with 1X TBST before Supersignal ™ chemiluminescence reagent (Thermo Scientific, Rockford, IL) was added and detected using Gel Doc™ XR ChemiDoc™ imaging system (BioRad, Hercules, CA) followed by quantification using ImageLab software (BioRad, Hercules, CA). Restore plus western blot buffer (Thermo Scientific, Rockford, IL) was used to strip membranes of antibodies prior to probing for other targets.

### Extracellular IGFBP-6 measurement

Media was collected 24hrs after treatment and stored at -20C until use. For ELISA measurements, cells were counted using a hemocytometer with trypan blue as a contrast. On average 1.5 million cells/well were counted. Measurement of secreted IGFBP-6 was done with an IGFBP-6 ELISA kit (Thermo Scientific, Rockford, IL) as described by the manufacturer. For immunoblot, media was concentrated using Amicon Ultra Centrifugal 3 kDa Filters (Millipore Sigma, St. Louis MO) as indicated by the manufacturer. Final protein concentration was determined by BCA assay (Thermo Scientific, Rockford, IL). 10-15 μg of protein were analyzed by immunoblot as described above.

### siRNA knockdown

Knockdown of IGFBP-6 was done in DMEM supplemented with 10% fetal bovine serum, 1mM sodium pyruvate, and 2mM L-glutamine (Life Technologies, Carlsbad, CA). Reverse transfections were done with lipofectamine RNAiMAX (Life Technologies, Carlsbad, CA). The following sequences were used to perform knockdowns: SiRNA 1 (Sense 5’-GGAGAAUCCUAAGGAGAGUtt-3’, Antisense 5’-ACUCUCCUUAGGAUUCUCCtc-3’), SiRNA 2 (Sense 5’-GAAUCCUAAGGAGAGUAAAtt-3’, Antisense 5’-UUUACUCUCCUUAGGAUUtc-3’). Silencer™ Negative control #1 (Life Technologies, Carlsbad, CA) was used as a negative control. For immunoblot, 250,000 cells per well were plated in a 6-well plate. After 48 hours, the media was switched to charcoal stripped DMEM containing a steroid treatment as described above.

### Cell viability assay

For cell viability, reverse transfection with 100,000 cells was performed. After 48 hours, the media was switched as described and cells were counted after 5 days. After 5 days of treatment with a steroid, cells were detached with trypsin and harvested in charcoal stripped DMEM. Cells were counted with a hemocytometer and compared to vehicle-treated samples. 0.4% Trypan blue (Life Technologies, Carlsbad, CA) was used as a contrast.

### Establishment of stably transfected cells

Human IGFBP-6 expression vector (NM_002178) and the control vector ORF were purchased from OriGene (Rockville, MD). Transfection was performed using Lipofectamine 3000 reagent in serum-free Opti-MEM (Life Technologies, Carlsbad, CA) according to the manufacturer’s protocol. After allowing transfection for 96 hours, cells were washed with 1X PBS, and allowed to recover in maintenance media for 24 hours then washed with 1X PBS followed by the addition of maintenance media containing 400 μg/mL Geneticin (Life Technologies, Carlsbad, CA). All stably transfected cells were validated after selection by immunoblot and RT-PCR. The stably transfected cells with an IGFBP-6 containing plasmid were named MCF7 BP6 and the control MCF7 EV.

### Statistical analysis

All statistical analyses were performed using R. Statistical analysis included ANOVA with Tukey’s *post-hoc* test. Differences were considered significant if p ≤ 0.05. All error bars are standard error of the mean.

## Results

### High IGFBP-6 expression is associated with better overall survival and recurrence free survival in PR+ breast cancers

**Figure 1:**
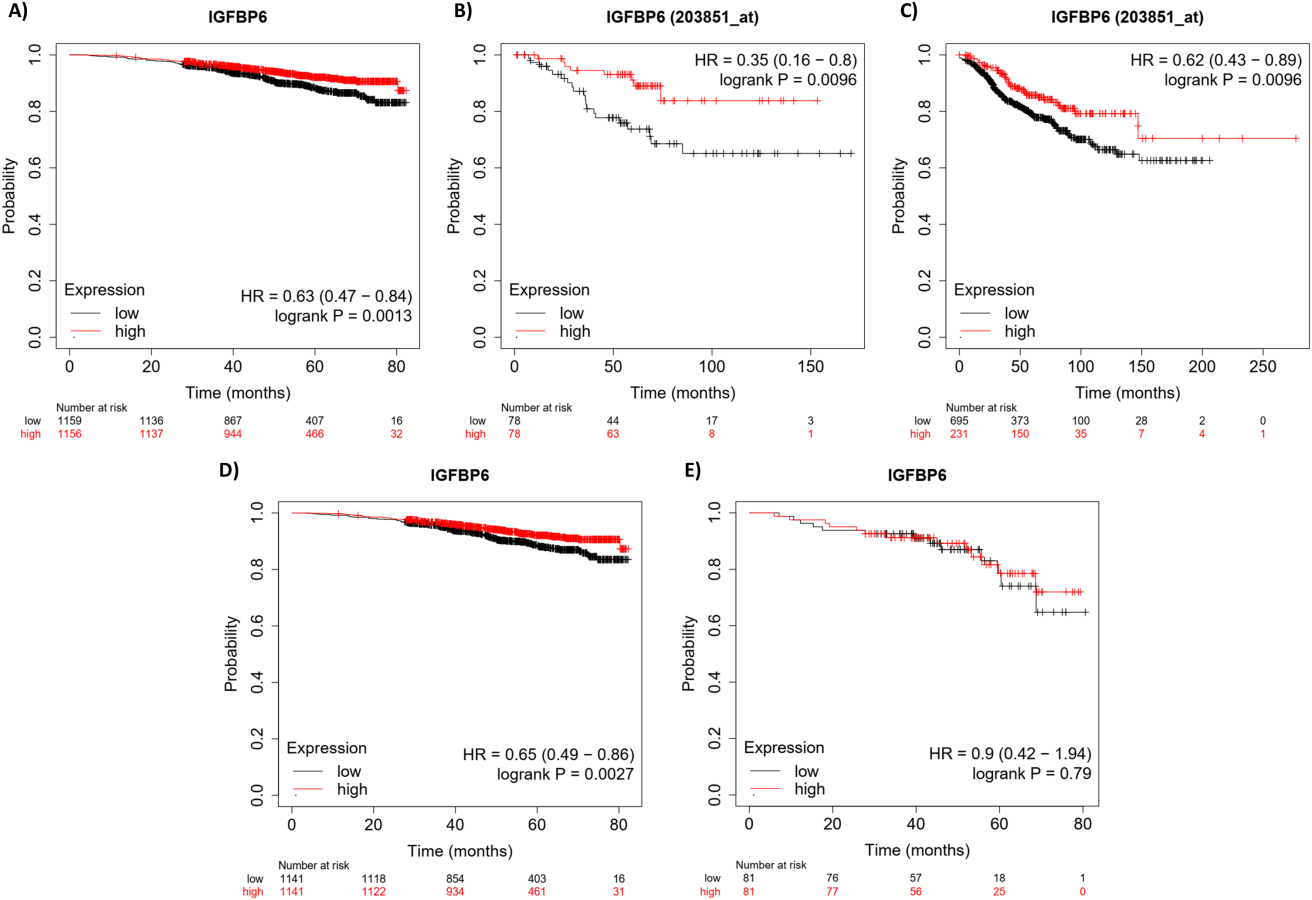
High IGFBP-6 expression is associated with better overall survival and recurrence free survival in R+ breast cancers. **(A)** Overall ival analysis using RNA-Seq data. PR positive patient data are separated by the median. **(B)** Overall survival analysis using GeneChip Data from GEO and EGA. PR positive patient data are again separated by the median. **(C)** Recurrence-free survival analysis using Gene chip data. PR positive patient data were separated by the upper quartile. **(D)** Overall survival analysis using RNA-seq data for ER positive and PR positive patients. Data is divided by the median. **(E)** Overall survival analysis using RNA-seq data for ER positive and PR negative patients. Data were divided by the median.

Previous studies have demonstrated that survival outcomes are improved by IGFBP-6 for patients with breast cancer (37). This study addressed ER positive breast cancers but did not determine if the protection was dependent on PR expression. Kaplan-Meier plots (**Figure 1**) were generated using the KM plotter tool with an RNA-seq dataset and a gene chip dataset from the gene expression omnibus (GEO) and the European Genome-Phenome Archive (EGA) (35). The RNA-seq data shows that patients with PR+ breast cancer had improved overall survival when IGFBP-6 was above the median in both data sets. For RNA-seq, the hazard ratio was 0.63 (**Figure 1A**) and for Gene-chip it was 0.35 (**Figure 1B**). Recurrence free survival was analyzed using gene-chip data (**Figure 1C**). Patients in the upper quartile of IGFBP-6 expression exhibited improved recurrence free survival with a hazard ratio of 0.62. These results indicate that high expression of IGFBP-6 is associated with improved survival in patients with PR+ breast cancers. Selecting patients that are both ER+ and PR+ results in a benefit in overall survival (**Figure 1D**). To determine whether the benefit conferred by IGFBP-6 is from ER or PR, patient data was selected from patients with ER+ and PR- breast cancers (**Figure 1E**). There was no significant improvement in overall survival from high IGFBP-6 in ER+, PR- breast cancer patients.

### IGFBP-6 is induced by progesterone in T47D breast cancer cells

**Figure 2:**
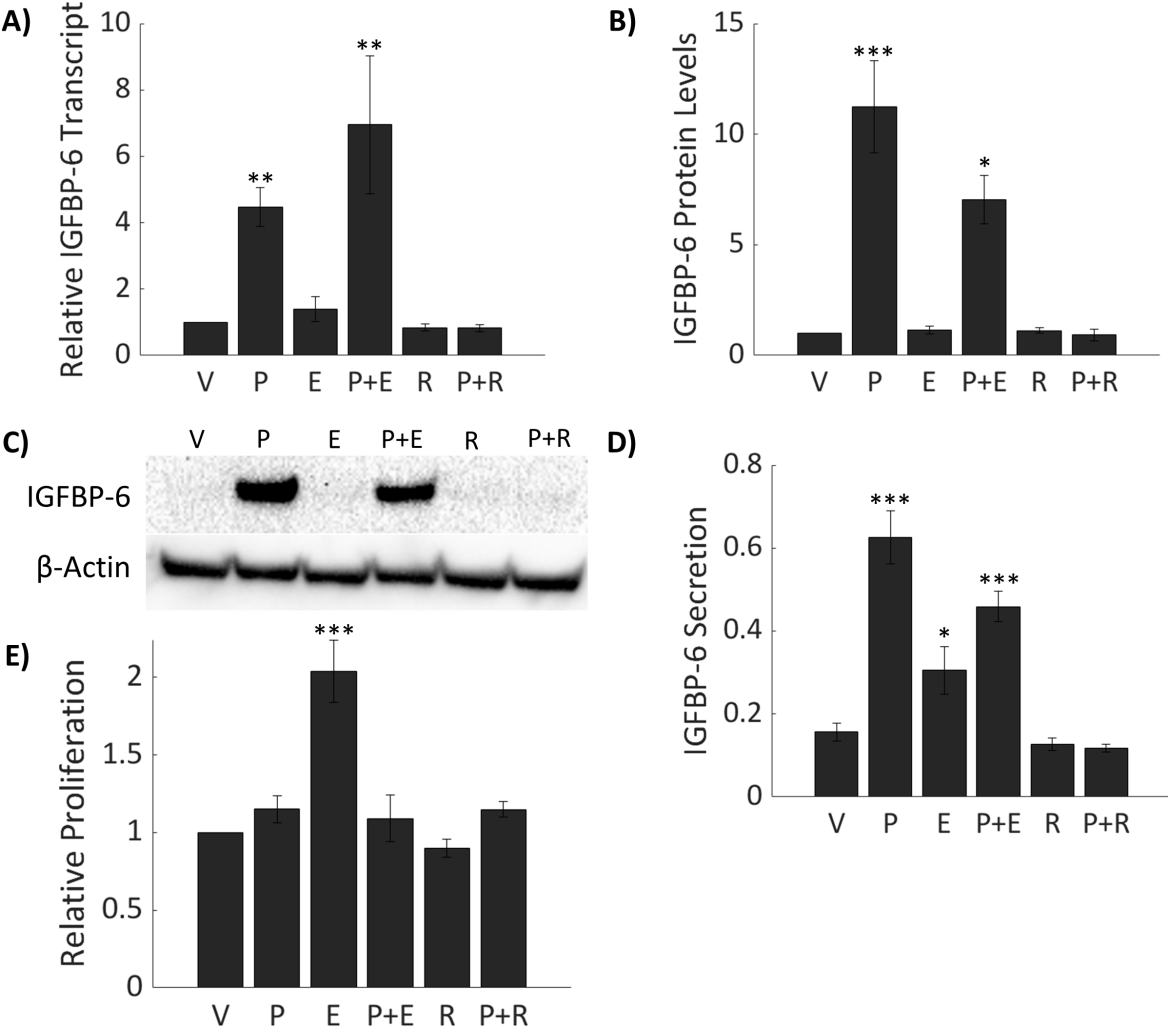
IGFBP-6 is induced by Progesterone (P4) in T47D breast cancer cells. Asterisks indicate significance relative to the vehicle. (* means p< 0.05, ** means p<0.01, and *** means p<0.001) **(A)** IGFBP-6 transcriptional expression with various steroid hormone and mifepristone treatments (p<0.0001). V stands for ethanol, P for progesterone, E for estradiol, and R for mifepristone (RU486). **(B)** IGFBP-6 protein levels follow transcriptional levels (p<0.0001). **(C)** Representative intracellular IGFBP-6 immunoblot. **(D)** IGFBP-6 secretion. Units are in fg/mL/cell. IGFBP-6 concentrations are normalized by cell count (p<0.01). **(E)** Relative proliferation of T47D cells in response to steroid hormones (p<0.0001).

Prior studies have demonstrated that myometrial IGFBP-6 gene expression correlates with plasma P4 concentrations in pregnant rats (38). To determine if P4 regulates IGFBP-6 expression in breast cancer, T47D cells were treated with 50nM P4. IGFBP-6 is induced transcriptionally 5-fold with P4 alone and 6.9-fold with cotreatment of 50nM P4 and 10nM estradiol (E2) (**Figure 2A**). Treatment with E2 alone produced no significant changes to IGFBP-6 transcript. A 25 nM dose of Mifepristone (RU 486) produced no significant changes in IGFBP-6 transcript but was sufficient to block induction of IGFBP-6. These results indicate that IGFBP-6 transcription is regulated by progesterone receptor activity. Analysis by immunoblot demonstrates that IGFBP-6 protein levels follow the same pattern as the transcript (**Figures 2B, C**). Intracellular IGFBP-6 induction was observed to peak by 24 hours and was sustained at 36 hours. Secretion of IGBP-6 was measured by ELISA and normalized to cell counts taken after media was collected (**Figure 2D**). On average, around 1.5 million cells were counted. An approximate 4-fold increase in IGFBP-6 secretion was induced produced by P4 while E2 treatment alone resulted in a 2-fold increase in secretion of IGFBP-6 compared to the vehicle and cotreatment with P4 and E2 produced a 3-fold increase in secretion. Treatment with RU-486 alone or 50 nM P4 in combination resulted in no change in IGFBP-6 secretion. Cell proliferation was also measured in the presence of steroid hormones (**Figure 2E**). P4 alone produces no difference in proliferation when compared to cells treated with the vehicle. Treatment with E2 alone produces an approximate two-fold increase in proliferation. However, co-treatment with P4 and E2 results in no increase in proliferation compared to the control. Treatment with RU-486 alone or in combination with P4 produced no significant changes in proliferation. These results demonstrate that intracellular and extracellular levels of IGFBP-6 are regulated by progesterone and suggest a role for IGFGP-6 in the observed antagonism.

### Knockdown of IGFBP-6 counteracts proliferative antagonism of progesterone

**Figure 3:**
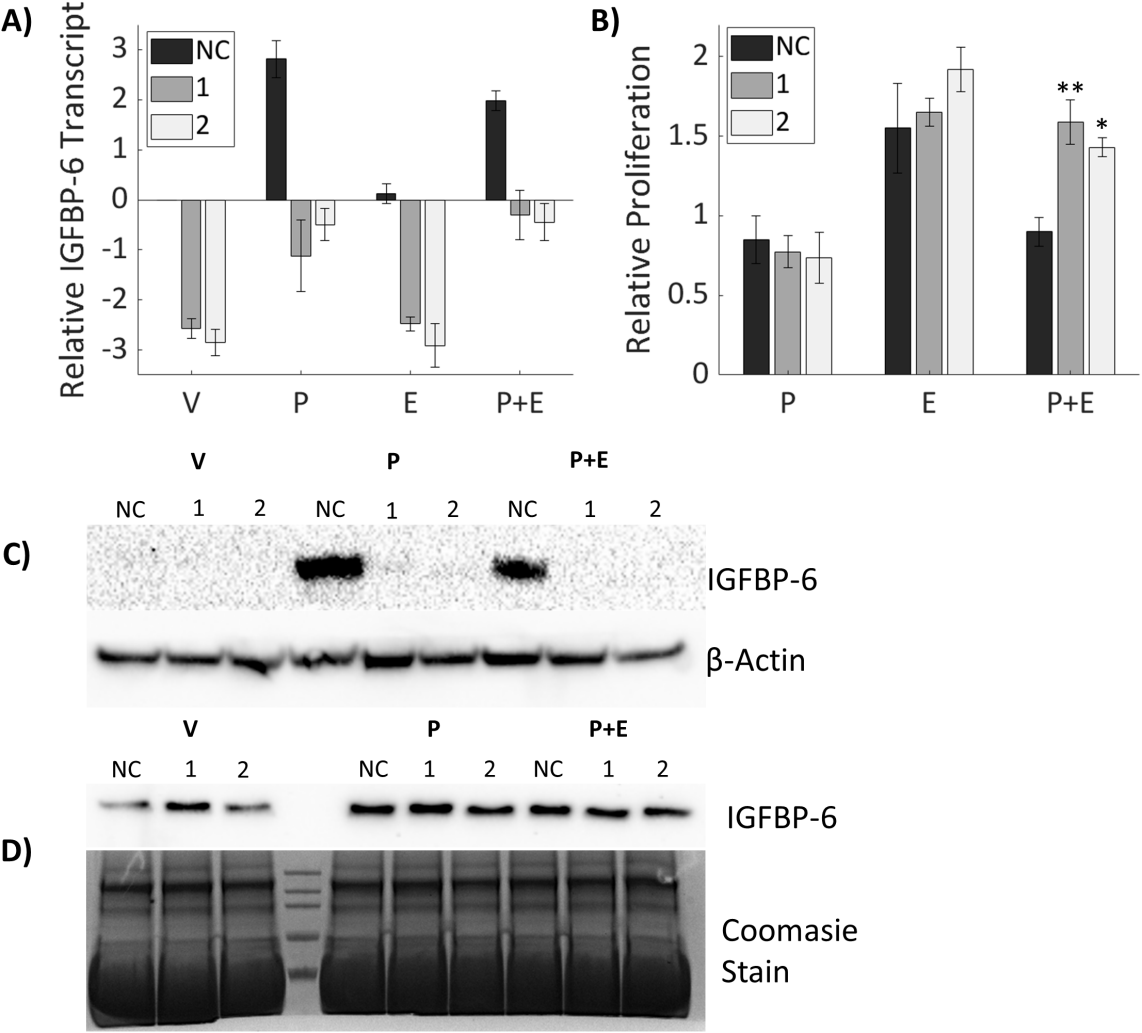
Knockdown of IGFBP-6 counteracts proliferative antagonism of progesterone. Asterisks indicate significance relative to the negative control for each steroid treatment. (* means p< 0.05, ** means p<0.01). **(A)** IGFBP-6 transcript following siRNA mediated knockdown. All values are log base 2 transformed. For the knockdown p< 0.0001, for steroid treatments p<0.0001, and the interaction was not significant. **(B)** Cell proliferation after IGFBP-6 knockdown. All values are relative to cells treated with the vehicle which was set to 1 for each siRNA. For the knockdown p<0.05, for steroid treatments p< 0.0001, and for the interaction p<0.01. **(C)** Representative intracellular IGFBP-6 immunoblot. **(D)** Representative extracellular IGFBP-6 immunoblot.

Since IGFBP-6 induction is associated with conditions in which cell proliferation is repressed it was hypothesized that IGFBP-6 has a role in slowing breast cancer cell proliferation. SiRNA knockdown was done to inhibit induction of IGFBP-6 in the presence of steroid hormones and mRNA levels were measured by RT-PCR. IGFBP-6 transcript decreased by at least 75% compared to the corresponding negative control and steroidal treatment (**Figure 3A**). Knockdown of IGFBP6 prior to treatment with P4 or P4 plus E2 blocked induction of IGFBP-6. IGFBP-6 levels after knockdown were not significantly different than the negative control plus vehicle. Immunoblot analysis results show an induction of intracellular IGFBP6 in the negative control with P4 but undetectable levels following knockdown (**Figure 3C**). Extracellular levels of IGFBP-6 remained stable despite knockdown (**Figure 3D**). This result suggests that any observed effects of IGFBP-6 knockdown may be attributed to loss of intracellular IGFBP-6 function.

To measure potential effect on cell proliferation, T47D cells were treated with SiRNA for 48 hours then switched to media containing P4, E2 or both for 5 days. Cell proliferation increased continually over the five days under all conditions. Relative proliferation was measured against the vehicle for each siRNA treatment. There were no significant differences in proliferation from the vehicle for either IGFBP-6 knockdown sequence. Cells treated with negative control behaved similarly to cells without IGFBP-6 knockdown. When treated with P4 or E2 alone, knockdown of IGFBP-6 resulted in no differences in cell proliferation. However, in cells where IGFBP-6 was knocked down, treatment with P4 plus E2 did not decrease proliferation (**Figure 3B**). These results demonstrate that IGFBP-6 modulates proliferative antagonism of E2 by P4.

### Knockdown of IGFBP-6 reduces progesterone receptor levels

**Figure 4:**
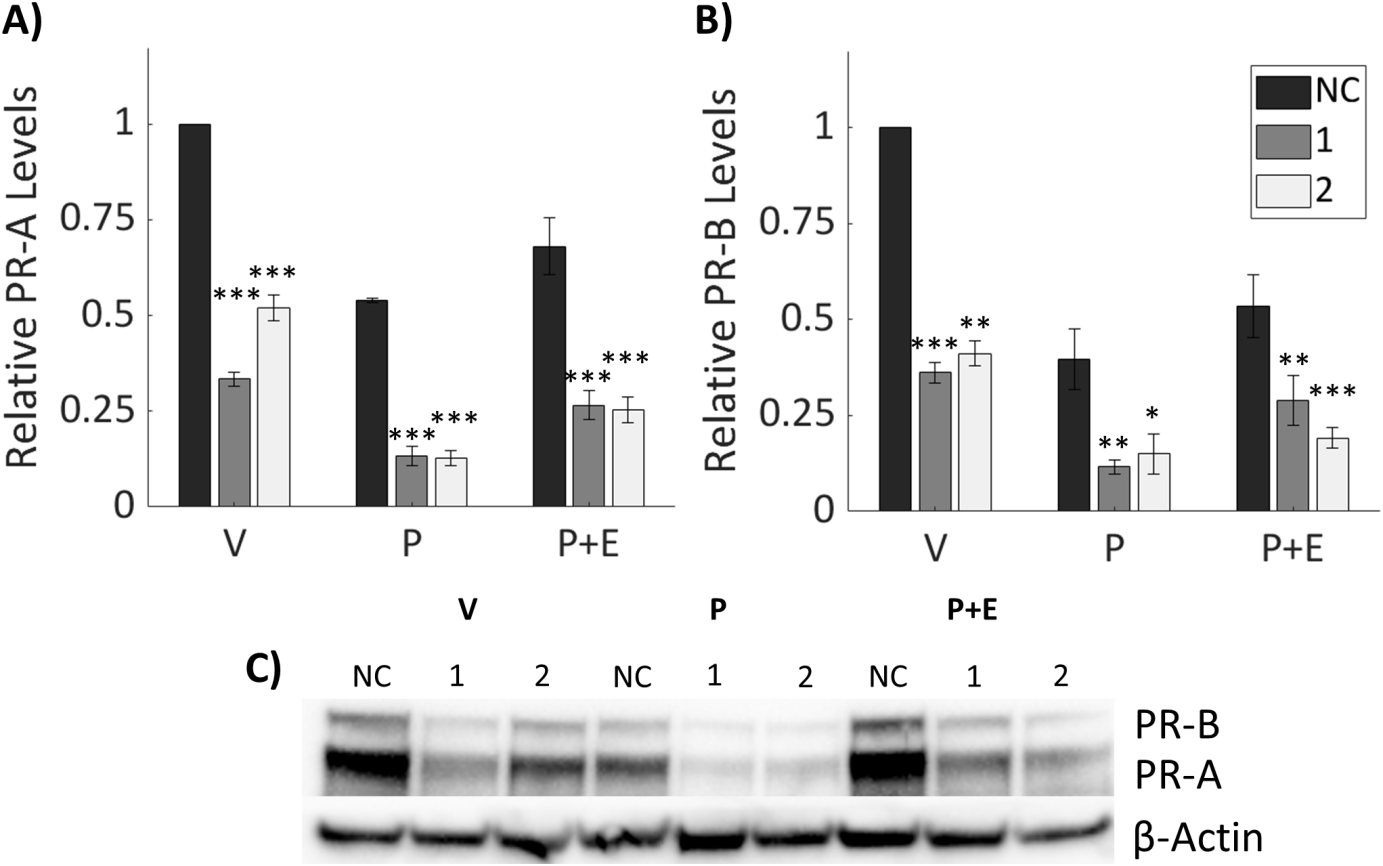
Knockdown of IGFBP-6 reduces Progesterone Receptor levels. Asterisks indicate significance relative to the negative control for each steroid treatment. (* means p< 0.05, ** means p<0.01, and *** means p<0.001). **(A)** PR-A levels decrease following IGFBP-6 knockdown. Statistical analysis was performed on Rank-Transformed data. For the knockdown p<0.0001, for the steroid treatments p<0.0001, and for the interaction p<0.05. **(B)** PR-B levels decrease following IGFBP-6 knockdown. For the knockdown p<0.0001, for the steroid treatments p<0.0001, and interactions were nonsignificant. **(C)** Representative immunoblot for PR-A and PR-B.

To explore possible mechanisms by which IGFBP-6 modulates inhibition of E2-induced proliferation by P4, PR levels were measured by immunoblot (**Figure 4**). Previous studies indicate that PR-A, the smaller isoform, is responsible for transpression of ER activity (25). Knockdown of IGFBP-6 decreased PR-A and PR-B levels relative to the negative control with every steroid treatment (**Figures 4A–C**). Treatment with the vehicle after IGFBP-6 knockdown reduced PR-B down to ~40% compared to the negative control. When treated with progesterone, PR-B decreased to ~40% in cells treated with the negative control. In the knocked down cells, progesterone levels decreased even further down to either 10 or 15%. Treatment with progesterone and estradiol decreased PR-B to 60% in cells treated with the negative control. With knockdowns, cotreatment with progesterone and estradiol decreased PR-B down to 24 or 17%. PR-A followed a similar trend. Treatment with the vehicle resulted in PR-A levels dropping to 37 or 52%. Progesterone decreased PR-A levels in the negative control to 54% and 11% or 13% in the knockdowns. Cotreatment with progesterone and estradiol decreased PR-A levels to 68% in the negative control and ~25% in the knockdowns. Increased phosphorylation at S294 for PR-B or S130 for PR-A was expected to be increased as this site is phosphorylated by mitogen activated protein kinase (39). However, relative phosphorylation of PR-B (S294) and PR-A (S130) was unchanged by knockdown of IGFBP-6 (**Supplementary Figure S3**) meaning that increased activation was not leading to increases in PR degradation. Statistically, knockdown of IGFBP-6 and treatment with steroid hormones produced significant decreases in PR levels. These results indicate that that IGFBP-6 expression associates with PR levels in T47D cells and suggest that IGFBP-6 can enhance PR activity by stabilizing PR levels.

### Downstream progesterone signaling is modulated by knockdown of IGFBP-6

**Figure 5:**
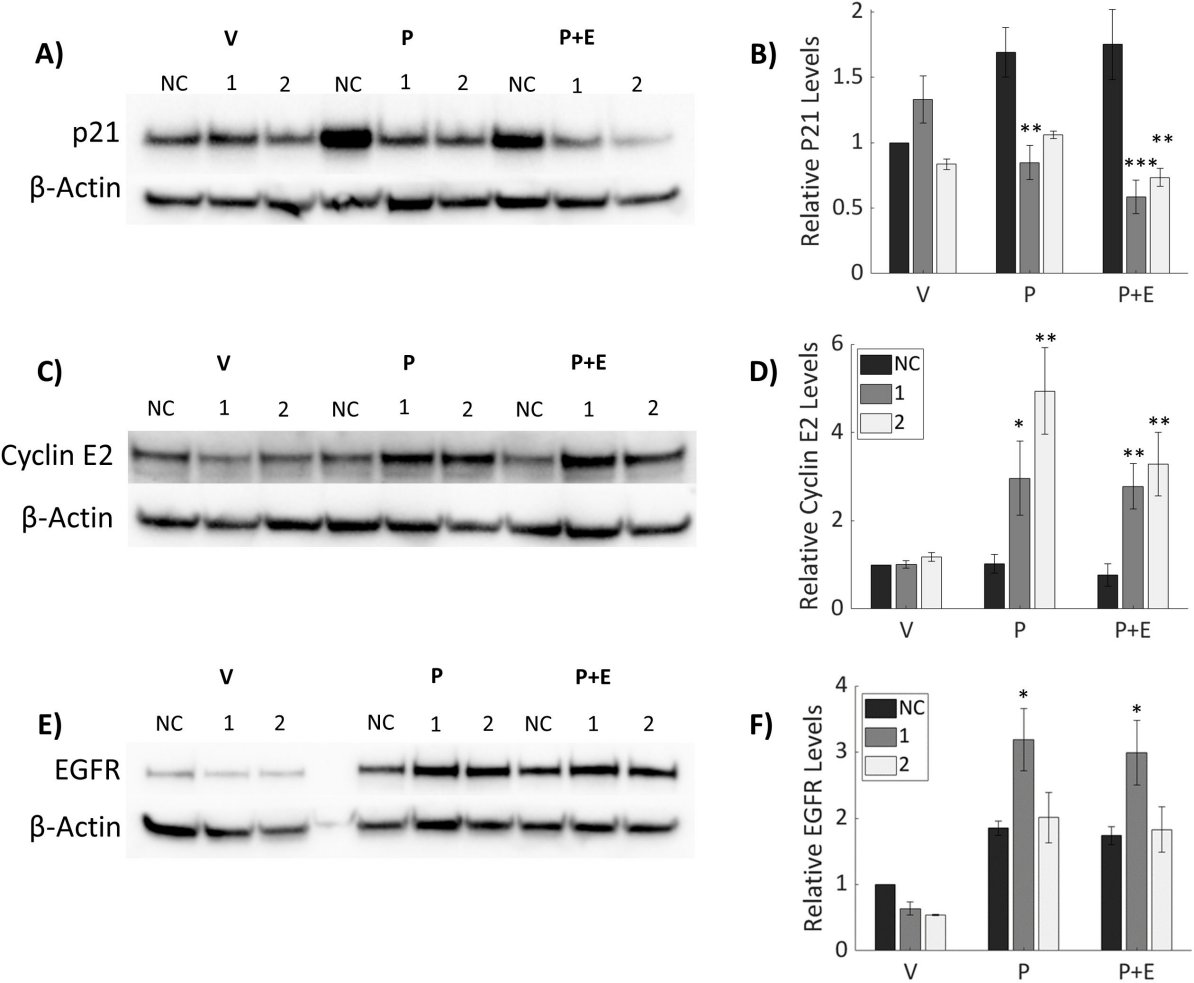
Downstream Progesterone signaling is modulated by knockdown of IGFBP-6. Progesterone signaling is dysregulated upon IGFBP-6 knockdown. Asterisks indicate significance relative to the negative control for each steroid treatment. (* means p< 0.05, ** means p<0.01, and *** means p<0.001). **(A)** Representative P21 immunoblot. **(B)** Relative P21 levels following IGFBP-6 knockdown. For knockdowns p< 0.0001, steroid treatments were nonsignificant, and for the interaction p<0.001. **(C)** Representative Cyclin E2 Immunoblot. **(D)** Relative Cyclin E2 levels following IGFBP-6 knockdown. Statistical analyses were performed on Rank-transformed data. For the knockdown p<0.0001, for steroid treatments p<0.001, and for interaction p<0.05. **(E)** Representative EGFR immunoblot. **(F)** Relative EGFR levels following IGFBP-6 knockdown. For knockdowns p< 0.01, for steroid treatments p< 0.0001, and for the interaction p<0.05.

Downstream targets of P4 were analyzed (**Figure 5**) to determine the impact of IGFBP-6 knockdown on progesterone signaling as PR-B is responsible for most of the transcriptional activity between both isoforms of PR (23). As such, it was hypothesized that decreases in PR-B levels would result in decreased expression of target genes and subsequent protein expression induced by progesterone. Epidermal Growth Factor Receptor (EGFR) and p21, a cell cycle inhibitor, are induced by progesterone in T47D cells (13, 23). Treatment with P4 or P4 plus E2 resulted in an induction of p21 in the scrambled SiRNA control (**Figures 5A, B**). However, knockdown of IGBP-6 resulted in no induction of p21 in response to P4 or cotreatment with P4 and E2. EGFR is known to promote proliferation and survival in breast cancer (27). EGFR levels decrease slightly when treated with the vehicle following IGFBP-6 knockdown but exhibit siRNA-specific effects in presence of P4 (**Figures 5E, F**). SiRNA 1 increases EGFR levels while 2 has no change relative to the negative control. These results demonstrate that changes in EGFR levels do not account for the changes in proliferation seen in **Figure 3**. Rather p21 levels decrease which allows E2 to promote proliferation.

Progestogens are known drivers of cell cycle progression in breast cancer (40). P21 is an inhibitor of cell cycle progression, so proteins associated with cell cycle progression were analyzed. Cyclin E2 levels were elevated following P4 treatment in knocked down cells (**Figures 5C, D**). P4 did not induce either Cyclin E2 in cells treated with the scrambled SiRNA control. These results suggest that IGFBP-6 plays a role in suppressing Cyclin E2 by sustaining PR-A levels. Taken together, IGFBP-6 suppresses proliferation by regulating p21 and Cyclin E2 levels in response to P4.

### Stable IGFBP-6 over-expression increases EGFR levels in response to hormonal treatments

**Figure 6:**
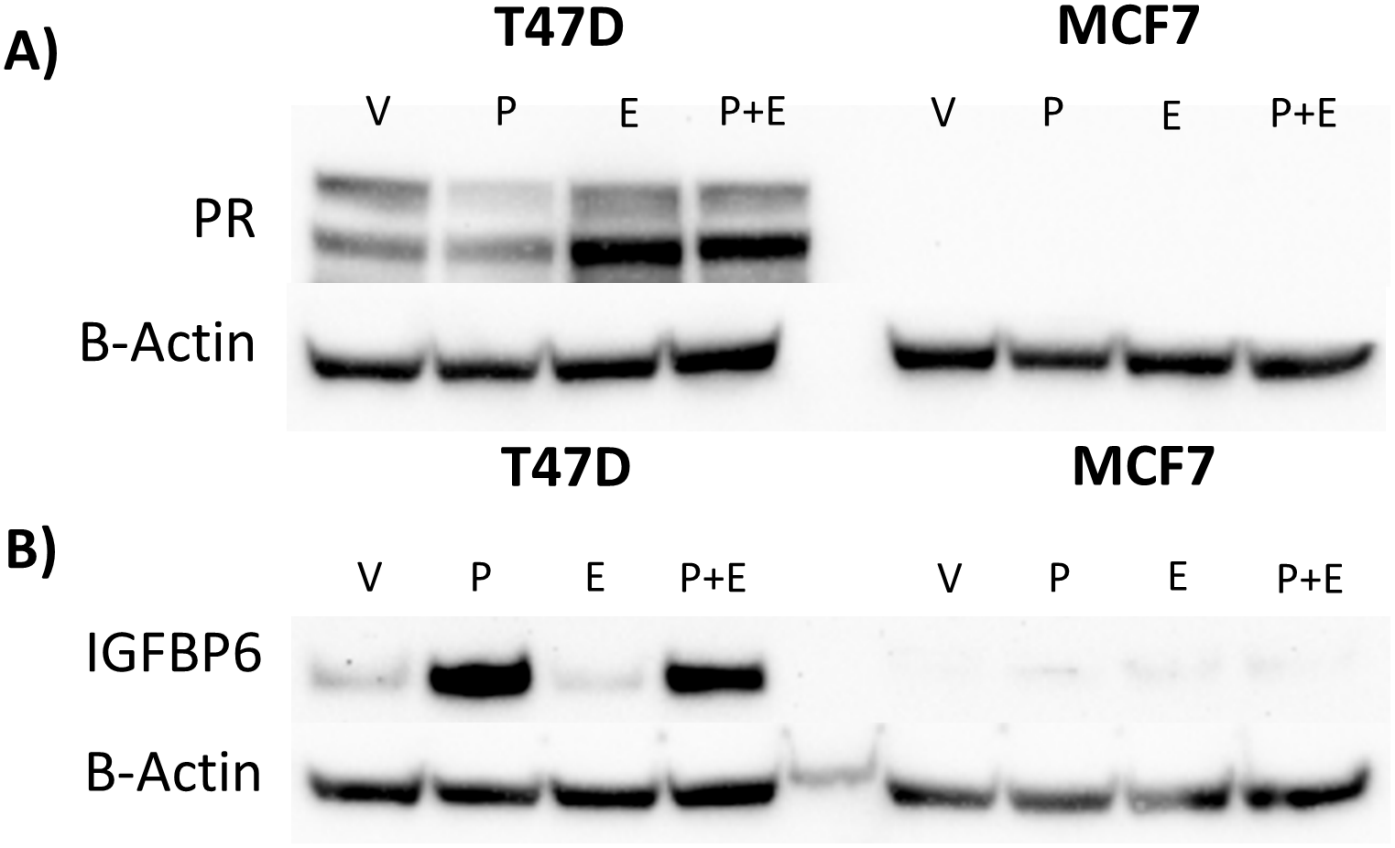
High IGFBP-6 levels are associated with PR levels in Hormone Receptor Positive Cells Lines. **(A)** Comparison of PR levels in T47D and MCF-7 cells. **(B)** Comparison of IGFBP-6 levels in T47D and MCF-7 Cells in response to P4 and E2.

**Figure 7:**
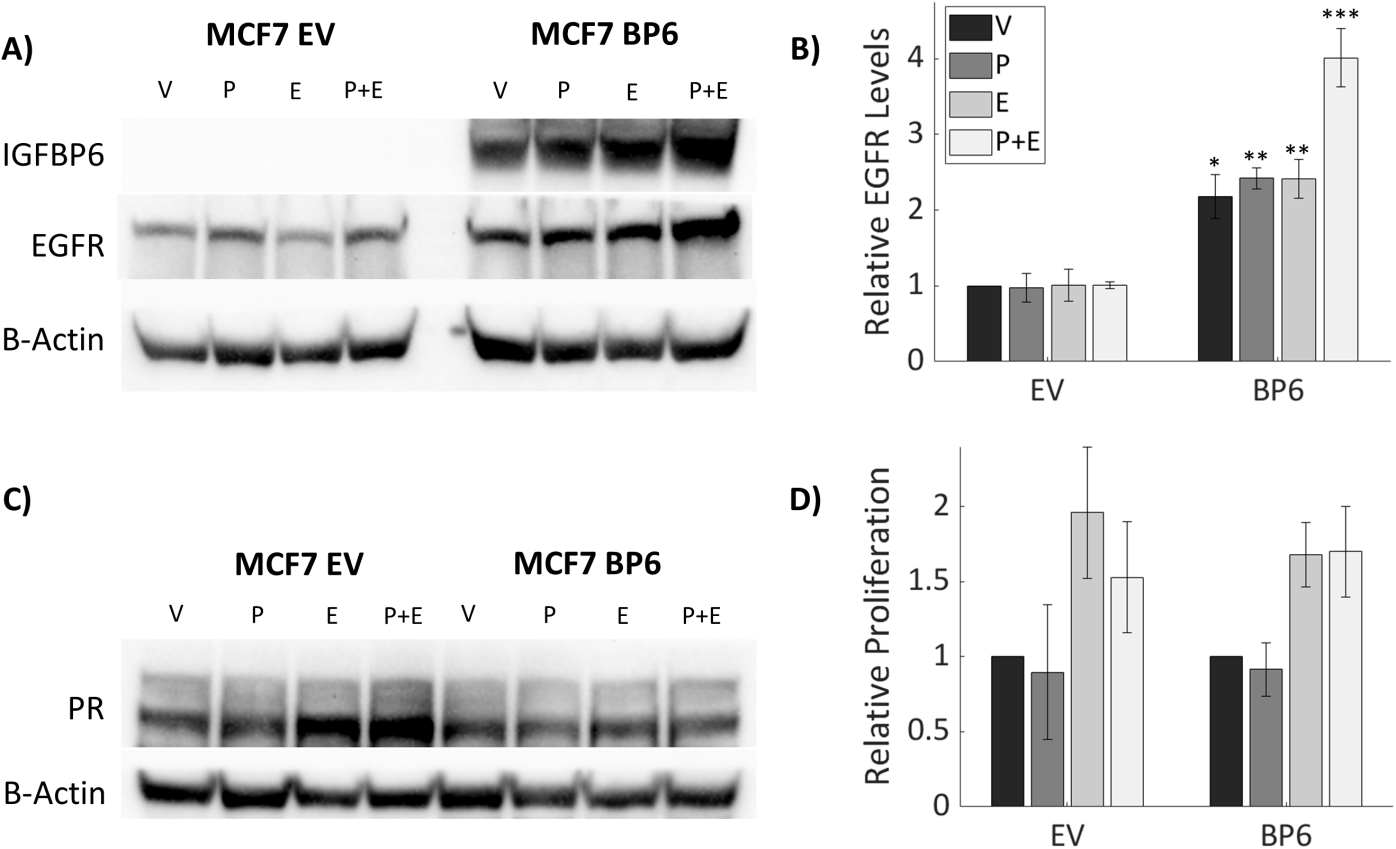
Stable IGFBP-6 Over-expression increases EGFR levels in response to hormonal treatments. Asterisks indicate significance between EV and BP6 cells for each steroid treatment. (* means p< 0.05, ** means p<0.01, and *** means p<0.001). **(A)** Stable overexpression of IGFBP-6 increases EGFR levels in MCF-7 cells. **(B)** Relative EGFR levels are increased with IGFBP-6 over-expression. EGFR levels increase even higher following estradiol plus progesterone treatment. P<0.001 for each cell type, p < 0.01 for the steroid treatments, and p <0.01 for the interaction. **(C)** PR levels following IGFBP-6 stable overexpression in MCF-7 cells. **(D)** Cell Proliferation following steroid hormone treatments in stable MCF-7 cells.

When compared to MCF-7 cells, T47D cells express higher levels of PR and IGFBP-6 (**Figure 6A**). It was observed that IGFBP-6 levels are not significantly changed upon treatment with P4,E2 or both (**Figure 6B**). To study the effects of IGFBP-6 in cells with endogenously low levels of PR-B and PR-A, MCF-7 cells were stably transfected with a plasmid to overexpress human IGFBP-6 (MCF-7 BP6) and a control plasmid (MCF-7 EV). MCF-7 BP6 cells have elevated levels of IGFBP-6 compared to MCF-7 EV (**Figure 7A**). PR-A, PR-B and EGFR levels were measured in response to steroid hormones in these cells. EGFR was not increased in MCF-7 EV cells upon treatment with P4 and/or E2 (**Figure 7B**). Increased EGFR was observed in MCF-7 BP6 cells compared to the MCF-7 EV cells and was increased 4-fold upon treatment with P4 and E2 but not with treatment with P4 or E2 alone. PR levels in the MCF-7 BP6 were not increased suggesting that IGFBP-6 may modulate PR actions (**Figure 7C**). Despite increases in EGFR, MCF7 BP6 cells do not exhibit any changes in proliferation compared to MCF7 EV cells in response to steroid hormone treatments (**Figure 7D**). Survival analysis (**Figures 1D, E**) demonstrates that ER positive, PR negative breast cancers have no benefit in overall survival from IGFBP6. The results obtained from MCF-7 cells modified to overexpress IGFBP-6 demonstrate that the observed protective effect of IGFBP-6 in ER-positive breast cancer is dependent on adequate PR expression and further supports our conclusion that IGFBP-6 expression improves overall survival in breast cancer patients via a PR-dependent mechanism.

## Discussion

Our results indicate that IGFBP-6 is induced by progesterone in T47D breast cancer cells and stabilizes progesterone receptor levels. Progesterone antagonizes the proliferative effects of E2 *in vitro* and this antagonism is abrogated upon knockdown of IGFBP-6. P21 is induced by P4 but this induction is blocked when IGFBP-6 is knocked down. Furthermore, cyclin E2 is not induced by progesterone except when IGFBP-6 was knocked down. These results demonstrate that IGFBP-6 alters responsiveness to P4 in T47D cells. Kaplan Meier analysis indicates that high IGFBP-6 expression is associated with improved outcomes for patients with PR+ breast cancer. The results presented suggest that this benefit is associated with PR actions. IGFBP-6 provides no significant benefit in ER+ but PR- breast cancers suggesting that IGFBP-6 provides a benefit when PR is high.

The IGFBPs are best known for being secreted proteins which attenuate IGF binding (1). SiRNA knockdown of IGFBP-6 resulted in a decrease in intracellular protein levels, but extracellular levels were unchanged. The charcoal stripped serum we used contains bovine IGFBP-6 which could not be distinguished from human IGFBP-6 as these proteins have high homology. Since the knockdown blocks induction of IGFBP-6 by P4, it was concluded that intracellular IGFBP-6 stabilizes PR levels, but further study is needed to determine a mechanism. IGF1R is known to promote mitogenic signaling through MAPK upon binding IGF-2 (4). IGF-2 is transcriptionally downregulated in response to P4 (**Supplementary Figure S1**). Additionally, MAPK phosphorylates PR at S294 (39, 41) but changes in MAPK phosphorylation were inconsistent between siRNA treatments. Alterations in phosphorylation at S294 were suspected but no changes in relative PR phosphorylation were observed in response to knockdowns (**Supplementary Figure S2**). PR has other post-translational modifications such as ubiquitination and sumoylation but further study is needed to determine if IGFBP-6 affects these post translational modifications (41, 42).

Progesterone signaling was altered following knockdown of IGFBP-6. EGFR levels were slightly decreased following knockdown and treatment with the vehicle. However, EGFR levels were unaffected by the knockdown in response to P4 or P4 plus E2. It should be noted that the difference between the vehicle and treatment with progesterone was increased when IGFBP-6 was knocked down. P21 was another protein induced by P4 in T47D cells. Knockdown of IGFBP-6 resulted in no significant induction of P21 in response to progesterone. However, when knocked down cells were treated with P4 and E2, a statistically significant decrease in P21 levels was observed. This demonstrates that IGFBP-6 regulates P21 levels in response to P4. P21 (Cip1/Waf1) is an inhibitor of the cell cycle by binding to Cyclin E/CDK2 complex and has been demonstrated to be a universal inhibitor of cyclin/CDK complexes (43).

IGFBP-6 knockdown produced cells with high or unchanged EGFR and low p21 in response to progesterone. High EGFR expression and low p21 expression is associated with worse prognosis in breast cancer patients (Zohny et al., 2018). Both p21 and EGFR are transcriptionally induced through the actions of the SP1 promoter (26). The findings presented in this study suggest that IGFBP-6 is a regulator of the protective effects of progesterone. Cyclin E2 was induced by P4 in knocked down cells. No induction was observed in the negative control in response to P4 or E2 suggesting that IGFBP-6 promotes the antiproliferative effects of P4. Cyclin E2 promotes the transition from the G1 to the S phase of the cell cycle where cells commit to division (28). One possible explanation for our observations is that IGFBP-6 enhances the repressive effects of PR-A. Further experimentation would be required to establish a molecular mechanism.

MCF-7 cells have very low levels of PR and IGFBP-6 compared to T47D cells. No induction of IGFBP-6 was observed in MCF-7 cells in response to progesterone. When IGFBP-6 is stably overexpressed in MCF-7 cells, an increase in EGFR was observed especially when treated with both P4 and E2. There was no increase in progesterone receptor in the IGFBP-6 overexpressing cells indicating that IGFBP-6 may modulate PR activity post-translationally. P21 was not affected by IGFBP-6 overexpression in MCF-7 cells.

IGFBP-6 has roles which are cell specific. In some cancers, IGFBP-6 promotes proliferation whereas in others it represses proliferation (1). The results presented demonstrate that IGFBP-6 is a tumor suppressor gene in hormone receptor positive breast cancers. By knocking down IGFBP-6, proliferative antagonism of progesterone was diminished in the presence of estradiol. Further study is needed to better define a mechanism which accounts for the exact function of IGFBP-6 in response to progesterone and the hypothesized stabilization of PR by IGFBP-6.

## Data Availability

The original contributions presented in the study are included in the article/**Supplementary Material**. Further inquiries can be directed to the corresponding author.
