# Supplementary material for: Insulin-like growth factor binding protein-6 modulates proliferative antagonism in response to progesterone in breast cancer
Source: Front Endocrinol (Lausanne). 2024 Dec 4;15:1450648. doi: 10.3389/fendo.2024.1450648 (PMC11652171; doi:10.3389/fendo.2024.1450648)
Supplement: Supplementary file 1 [file Presentation1.pptx]

## Slide 1
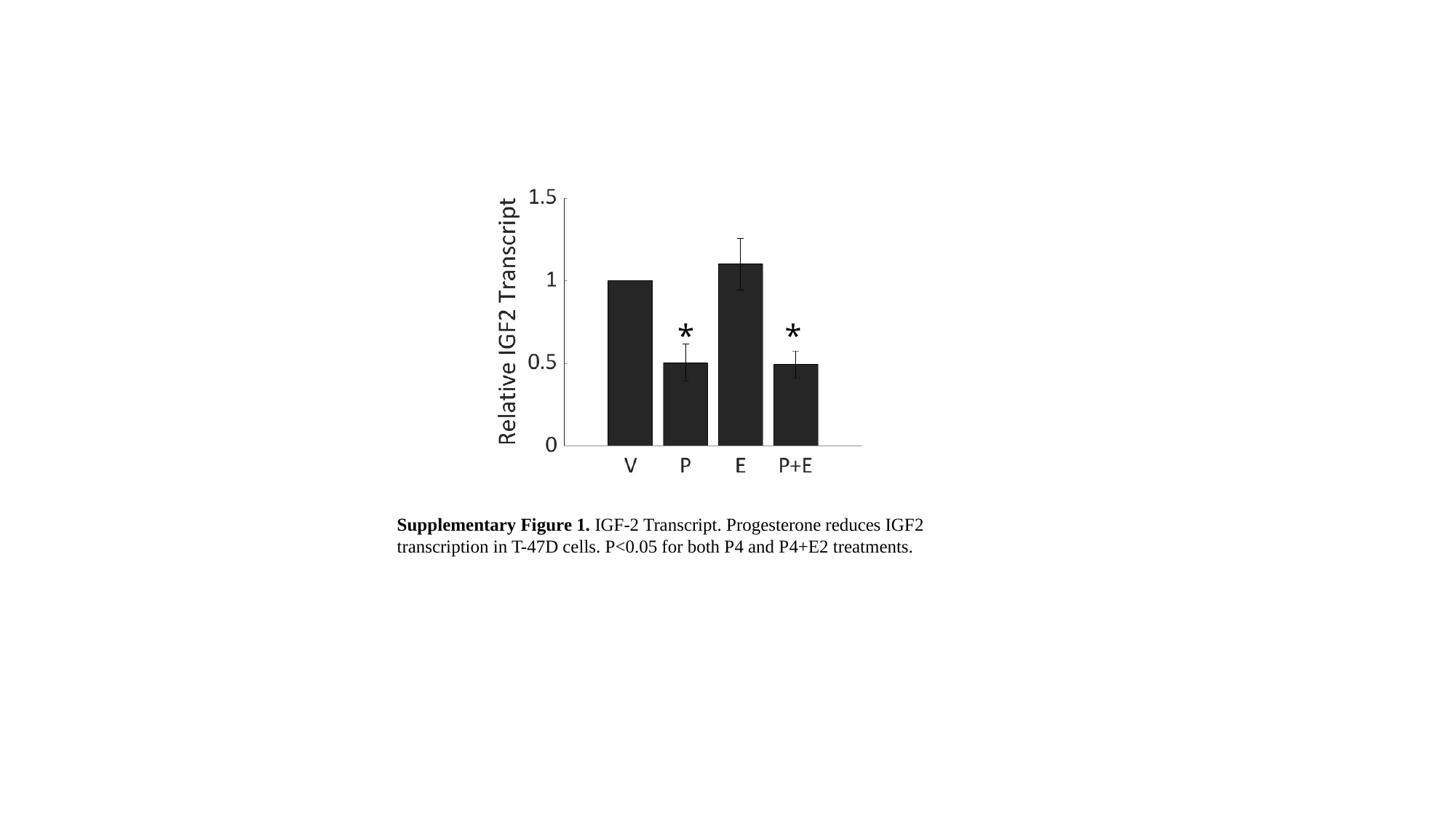

*
*
Supplementary Figure 1. IGF-2 Transcript. Progesterone reduces IGF2 transcription in T-47D cells. P<0.05 for both P4 and P4+E2 treatments.

## Slide 2
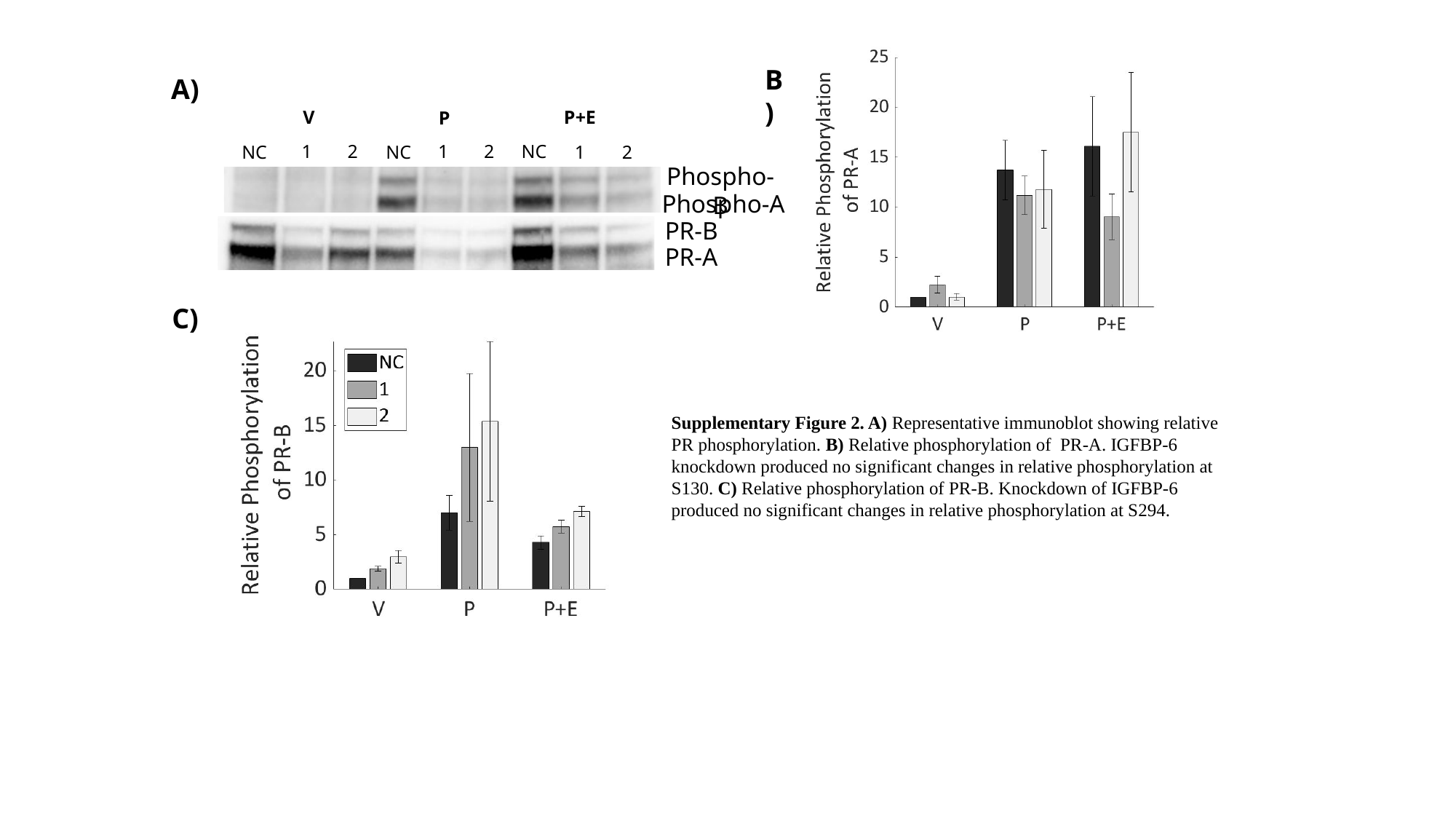

B)
A)
V
P+E
P
2
NC
1
1
2
NC
2
1
NC
Phospho-B
Phospho-A
PR-B
PR-A
C)
Supplementary Figure 2. A) Representative immunoblot showing relative PR phosphorylation. B) Relative phosphorylation of PR-A. IGFBP-6 knockdown produced no significant changes in relative phosphorylation at S130. C) Relative phosphorylation of PR-B. Knockdown of IGFBP-6 produced no significant changes in relative phosphorylation at S294.

## Slide 3
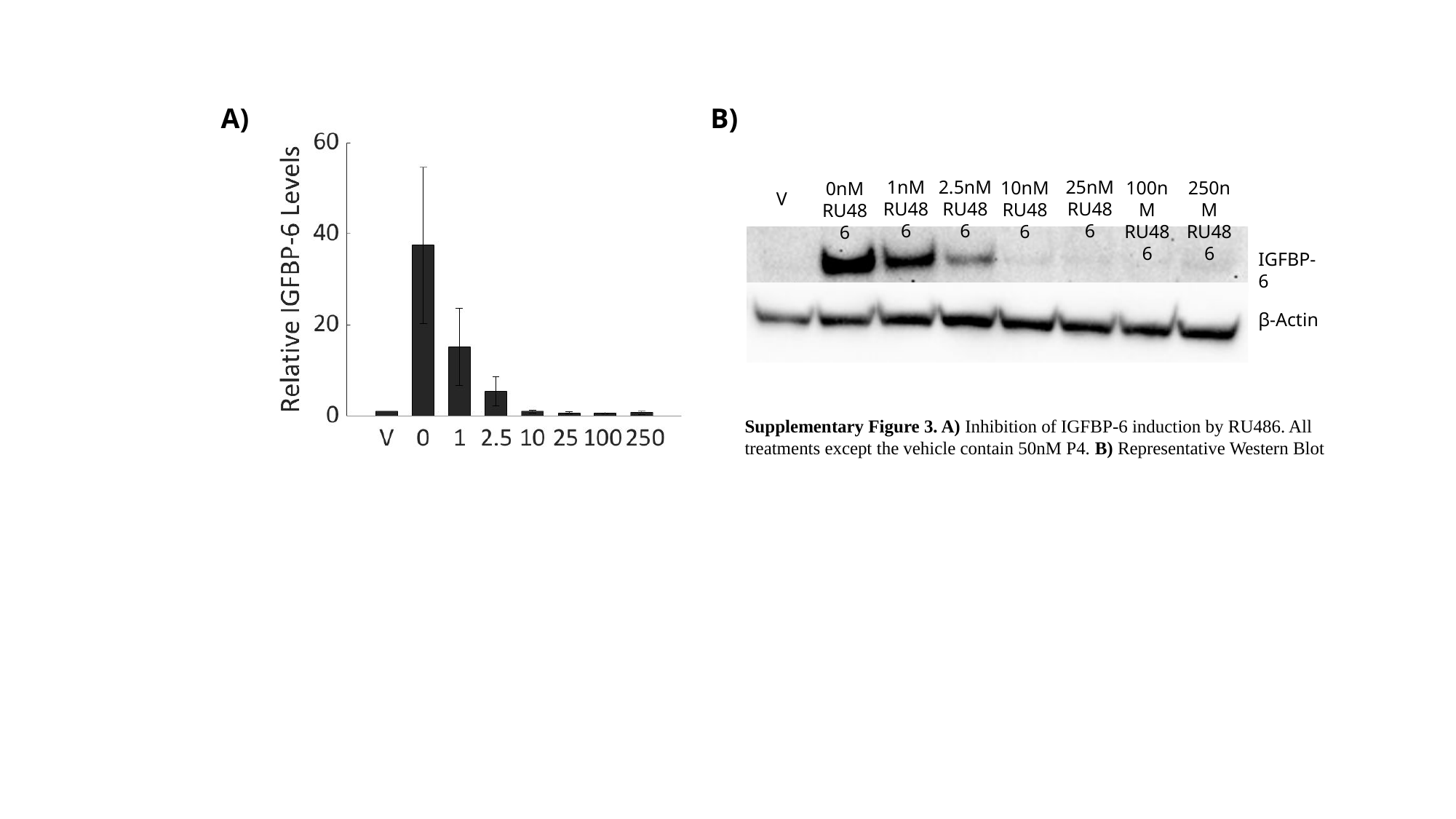

A)
B)
1nM RU486
2.5nM RU486
25nM RU486
10nM RU486
100nM RU486
250nM RU486
0nM RU486
V
IGFBP-6
β-Actin
Supplementary Figure 3. A) Inhibition of IGFBP-6 induction by RU486. All treatments except the vehicle contain 50nM P4. B) Representative Western Blot

## Slide 4
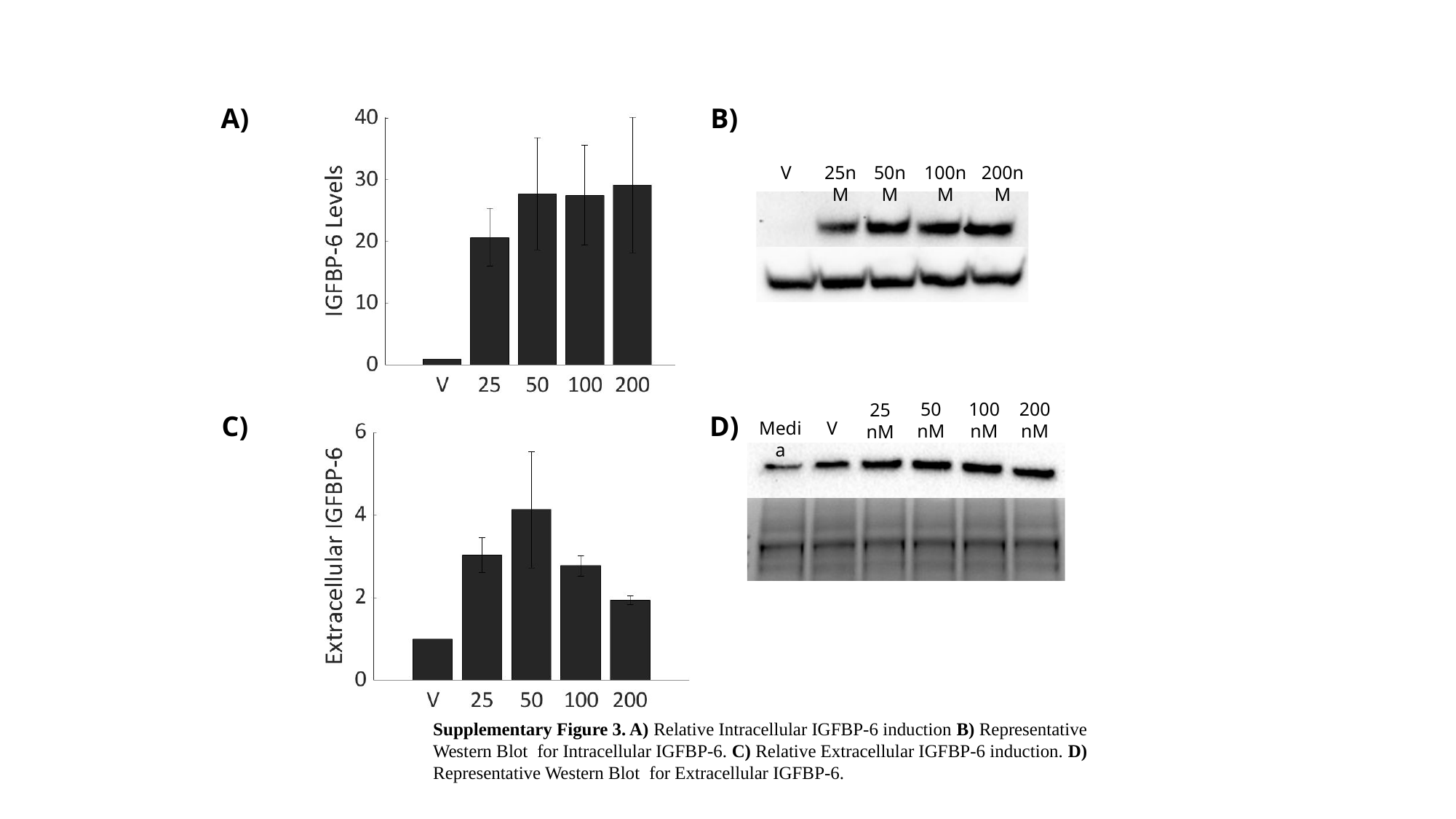

A)
B)
200nM
50nM
100nM
25nM
V
50
nM
100
nM
200
nM
25
nM
C)
D)
Media
V
Supplementary Figure 3. A) Relative Intracellular IGFBP-6 induction B) Representative Western Blot for Intracellular IGFBP-6. C) Relative Extracellular IGFBP-6 induction. D) Representative Western Blot for Extracellular IGFBP-6.
